# Minimum concentration of Amphotericin B in serum according to the formulation, dose, and daily or prolonged intermittent therapeutic regimen

**DOI:** 10.1590/0037-8682-0463-2018

**Published:** 2020-02-07

**Authors:** Leticia Aparecida Schiave, Erika Nascimento, Gilberto Gambero Gaspar, Fernando Crivelenti Vilar, Edson Zangiacomi Martinez, Cristiane Masetto de Gaitani, Roberto Martinez

**Affiliations:** 1 Universidade de São Paulo, Faculdade de Medicina de Ribeirão Preto, Departamento de Clínica Médica, Ribeirão Preto, SP, Brasil.; 2 Universidade de São Paulo, Faculdade de Medicina de Ribeirão Preto, Departamento de Medicina Social, Ribeirão Preto, SP, Brasil.; 3 Universidade de São Paulo, Faculdade de Ciências Farmacêuticas de Ribeirão Preto, Ribeirão Preto, SP, Brasil.

**Keywords:** Amphotericin B, Pharmacokinetics, Intermittent use, Ambulatory therapy, Cryptococcal meningitis

## Abstract

**INTRODUCTION:**

The therapeutic efficacy of daily amphotericin B infusion is related to its maximum concentration in blood; however, trough levels may be useful in intermittent regimens of this antifungal drug.

**METHODS:**

High performance liquid chromatography (HPLC) was used to determine the minimum concentration (Cmin) of amphotericin B in the serum of patients receiving deoxycholate (D-Amph) or liposomal amphotericin B (L-AmB) for the treatment of cryptococcal meningitis (n=28), histoplasmosis (n=8), paracoccidioidomycosis (n=1), and leishmaniasis (n=1).

**RESULTS:**

Daily use of D-Amph 30 to 50 mg or L-AmB 50 mg resulted in a similar Cmin, but a significant increase ocurred with L-AmB 100 mg/day. The geometric mean Cmin tended to decrease with a reduction in the dose and frequency of intermittent L-AmB infusions: 357 ng/mL (100 mg 4 to 5 times/week) > 263 ng/mL (50 mg 4 to 5 times/week) > 227 ng/mL (50 mg 1 to 3 times/week). The impact on Cmin was variable in patients whose dose or therapeutic scheme was changed, especially when administered the intermittent infusion of amphotericin B. The mean Cmin for each L-AmB schedule of intermittent therapy was equal or higher than the minimum inhibitory concentration of amphotericin B against *Cryptococcus* isolates from 10/12 patients. The Cmin of amphotericin B in patients with cryptococcal meningitis was comparable between those that survived or died.

**CONCLUSIONS:**

By evaluating the Cmin of amphotericin B, we demonstrated the therapeutic potential of its intermittent use including in the consolidation phase of neurocryptococcosis treatment, despite the great variability in serum levels among patients.

## INTRODUCTION

Amphotericin B is often the preferred treatment for patients with disseminated or complicated systemic fungal infections and visceral leishmaniasis. Amphotericin B is prepared as particles for intravenous use in a standard formulation complexed with deoxycholate or linked to lipids. These pharmaceutical presentations in form of particles and the intrinsic characteristics of the molecule are responsible for the peculiar pharmacokinetic properties of amphotericin B, including its ability to bind to blood lipoproteins and cell membranes, and its accumulation in phagocytic cells of the liver and spleen^1^. Owing to these characteristics and its slow excretion, liposomal amphotericin B (L-AmB) persists in blood for more than one week after infusion^2^, thereby attracting interest for its use in an intermittent, non-daily regimen for patients with low clinical severity^1^ ^,^ ^3^. An intermittent regimen facilitates the use of this antifungal antibiotic for ambulatory patients and has been used for prophylaxis and treatment consolidation of systemic fungal infections^4^ ^,^ ^5^.

As demonstrated using murine models of candidiasis and aspergillosis, maximum concentration in blood (Cmax) is the pharmacokinetic parameter that indicates the therapeutic efficacy of amphotericin B^6^ ^,^ ^7^. Although Cmax is reached shortly after the end of infusion, the levels of amphotericin B are significantly reduced after 24 to 48 h^8^. When amphotericin B is used at an interval of more than two days between infusions, determining the Cmin of the drug in blood immediately before the next infusion may be equally important. Further, the trough blood concentration can be compared to the minimum inhibitory concentration (MIC) of amphotericin B against the fungus causing the infection, thereby aiding in the maintenance or modification of the intermittent L-AmB scheme to achieve antifungal activity during the interval between infusions^9^.

In the present study, the Cmin of amphotericin B deoxycholate or L-AmB was determined in patients treated with different daily doses or in an intermittent regimen, which enabled us to compare the levels and evaluate the response of the individual whose therapeutic scheme was modified. The Cmin of amphotericin B in serum was compared to the MIC for *Cryptococcus* isolates from patients and the outcome of cryptococcal meningitis cases.

## METHODS

Thirty-seven adult patients treated with amphotericin B at the University Hospital of the Ribeirão Preto Medical School, Brazil were randomly included in the study. These patients had cryptococcal meningitis (n=27), histoplasmosis (n=8), paracoccidioidomycosis (n=1), and mucosal leishmaniasis (n=1), and 32 were coinfected with HIV. Patients were included in the study during the phase of induction and/or consolidation of treatment with amphotericin B, permitting a comparison of the blood levels of this antifungal agent in different therapeutic regimens. The 37 patients received a total dose of 620 mg to 10,250 mg (median = 2,870 mg) of amphotericin B in a therapeutic course that ranged from 10 days to 344 days (median = 39 days).

Initial treatment (daily doses) was carried out with amphotericin B deoxycholate (Anforin®, Cristália, Brazil) (D-Amph) in 3 cases, L-AmB (AmBisome®, United Medical, Brazil) in 17 cases, and D-Amph in the remaining 17 patients, followed by replacement with L-AmB due to the side effects of the deoxycholate formulation. For the D-Amph therapeutic plan, we considered the use of 50 mg/day, approximately 0.7 to 1.0 mg/kg weight/day; however, this daily dose was commonly reduced to 30 or 40 mg due to the nephrotoxicity of this formulation. L-AmB was used at doses of 50 to 200 mg/day, approximately 0.7 to 4 mg/kg weight/day, according to the medical decision and the availability of this medication. 

The regimen of intermittent L-AmB infusion was used during the consolidation phase of antifungal therapy, particularly in cases of cryptococcal meningitis. Generally, the dose and the frequency of infusion were progressively reduced as the patient improved and became stabilized. Three types of intermittent therapeutic schemes were analyzed for comparison of the trough blood concentration of lipossomal amphotericin B: 100 mg/day four to five times per week, 50 mg/day four to five times per week, and 50 mg/day one to three times per week. Sequential and individualized modifications of therapy with amphotericin B were adopted for many patients and involving the daily dose and/or the frequency of infusions.

The amphotericin B levels of 32 patients were evaluated in the initial phase of treatment (daily doses) with deoxycholate and/or liposomal formulation, 12 of which were included in two or three therapeutic schedule groups according to the medication and dose prescribed. Amphotericin B levels of 16 patients were evaluated in the consolidation phase of treatment with intermittent L-AmB according to the dose prescribed and the weekly frequency of infusions, 9 of which had Cmin results in more than one of the three therapeutic schedules analyzed. To compare individual response to changes in the therapeutic schedule, Cmin variation for the same patient was analyzed in the initial phase of the treatment with daily doses (n=11) and the consolidation phase when non-daily doses of amphotericin B (n=11) were administered.

The Cmin of amphotericin B in serum was determined in blood samples collected immediately before drug infusion, between 20 h and 7 days after the previous dose, according to the daily or intermittent drug infusion. The level of amphotericin B was measured in 1 to 27 (median=4) blood samples per patient, corresponding to the same or different therapeutic schemes with this antifungal antibiotic. For patients with two or more determinations of Cmin under the same therapeutic scheme, the mean was calculated to represent the amphotericin B level of the patient in that scheme.

Amphotericin B (D-Amph or L-AmB) concentration in serum was measured by high-performance liquid chromatography (HPLC) using a Shimadzu instrument (Shimadzu Corporation, Japan). The Phenomenex Gemini 5µ C18 110A (Phenomenex-Allcron, USA) and Ascentis C18 Supelguard® (Sigma-Aldrich, USA) columns were employed for chromatography, and detection was performed at 405 nm. Amphotericin B was previously extracted by adding acetonitrile to the sample, followed by dissolution of this antibiotic in a mobile phase consisting of acetate buffer and acetonitrile at a 60:40 (v/v) ratio. Amphotericin B (Sigma, USA) concentrations of 25 ng/mL to 5,000 ng/mL were used to plot the calibration curve^10^.

*Cryptococcus* spp. susceptibility to amphotericin B was evaluated in isolates from 12 patients by the broth microdilution method proposed by the Clinical Laboratory Standards Institute^11^.

To determine the relationship between the Cmin of amphotericin B administered daily and the outcome of the 23 patients with cryptococcal meningitis at one year following the initiation of antifungal therapy, patients were divided into two groups, namely Survival (n=16) and Death (n=7). Deaths were caused by complications owing to cryptococcosis and/or bacterial infections and 6/7 occurred during the first three months of treatment. Among the surviving patients, 15/16 received L-AmB. Of these, 12 were treated for different duration courses with a dose of 100 mg/day. In 9/16 patients in the Survival group, fluconazole was also administered for up to 14 days after diagnosis. In the Death group, 7/7 patients received L-AmB, 4/7 of whom were treated with a dose of 100 mg/day for variable courses lengths. In this group, fluconazole was also administered to 4/7 patients for up to 14 days after the diagnosis of cryptococcosis.

As some patients were included in more than one therapeutic schedule with daily or intermittent amphotericin B, a mixed linear regression model was used in the statistical analysis of Cmin levels. This test was also applied to analyze the responses of the same patients receiving different types of amphotericin B intermittent therapy. The concentrations of amphotericin B were transformed in a logarithmic scale owing to its right-skewed distribution. Consequently, the results of the model were presented as geometric means with their respective 95% confidence intervals (CI). Comparisons between geometric means were based on the differences in least squares mean. The assumption of normality of residuals was verified using normal probability plots. SAS (version 9) was used for data analysis. The Wilcoxon test (Prism Program, version 6) was employed to compare the Cmin of the same patients under daily treatment with two different amphotericin B therapeutic schedules. The Mann-Whitney test (Prism Program, version 6) was used to compare the Cmin of amphotericin B for cryptococcal meningitis patients who survived to those who died and analyze the MIC of amphotericin B for C*ryptococcus* spp. isolated from these patients. The significance level was set at 0.05.

The study was approved by the Research Ethics Committee of the University Hospital, Ribeirão Preto Medical School, University of São Paulo (protocol nº 4096/2012). All patients provided written informed consent prior to participating in the study.

## RESULTS

**FIGURE 1:**
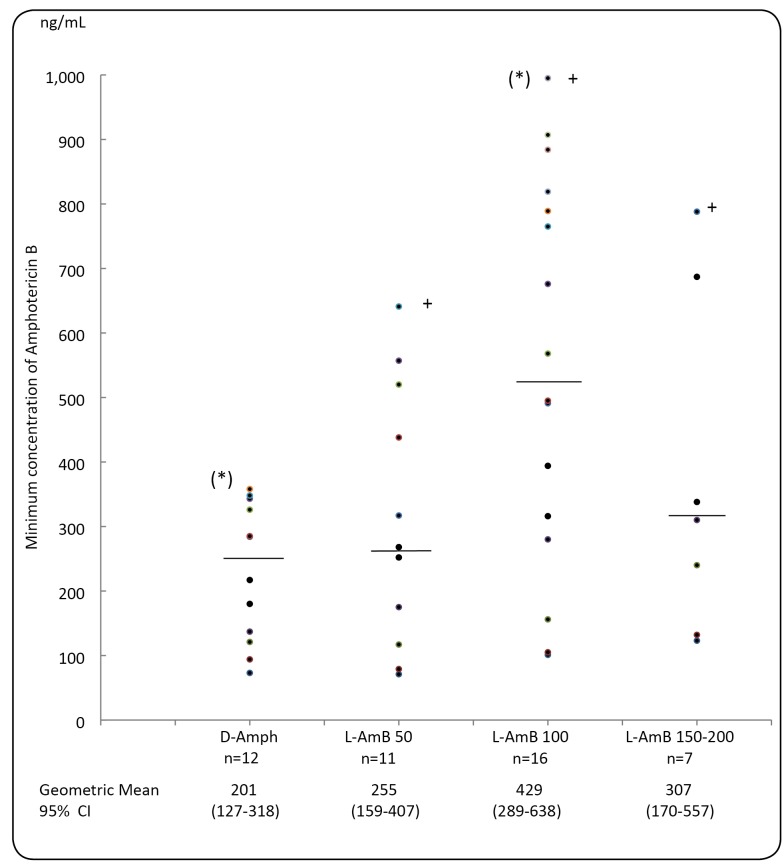
Minimum concentration of Amphotericin B in the serum (ng/mL) of patients receiving daily intravenous infusions of deoxycholate formulation (D-Amph) - 30 to 50 mg/day, and liposomal amphotericin B (L-AmB) - 50, 100, or 150 - 200 mg/day. (+) This value was reduced by 50% for graphic presentation. The median value is represented by a horizontal bar. (*) p< 0.01.

**FIGURE 2:**
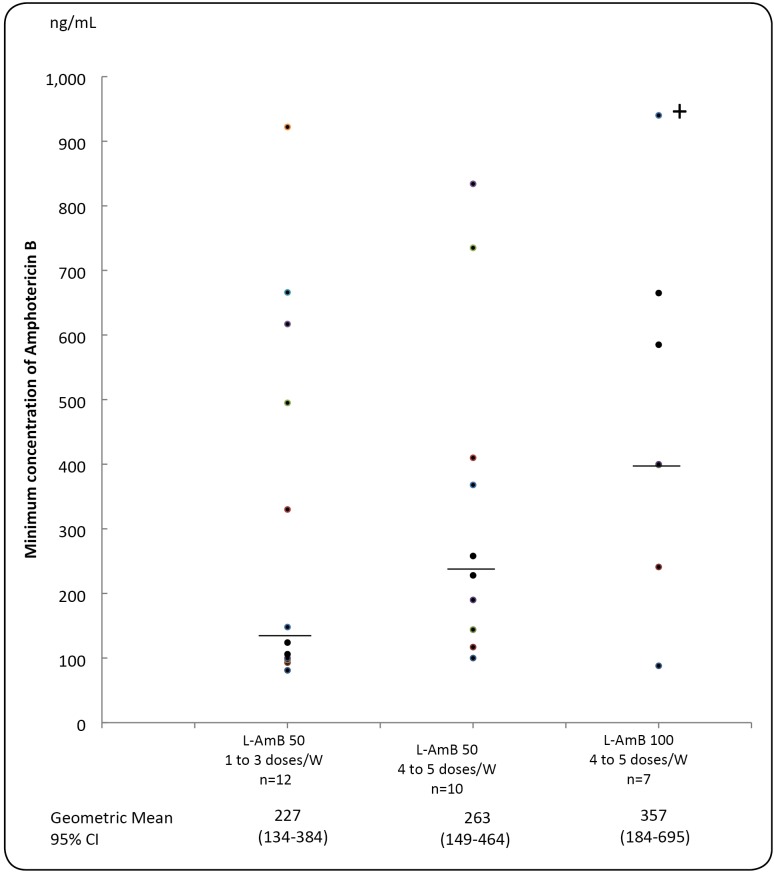
Minimum concentration of Amphotericin B in the serum (ng/mL) of patients receiving intermittent intravenous infusions of liposomal formulation (L-AmB) according to three therapeutic schedules: 50 mg, 1 to 3 times per week; 50 mg, 4 to 5 times per week, or 100 mg, 4 to 5 times per week. The median value is represented by a horizontal bar. (+) This value was reduced by 30% for graphical presentation.

In the daily infusion regimen of amphotericin B, median Cmin was found to be higher with L-AmB than D-Amph; however, the difference was only statistically significant between D-Amph 30 to 50 mg/day and L-AmB 100 mg/day (p˂0.01) (Figure 1). When L-AmB was used in the intermittent regimen, the geometrical mean of Cmin tended to be higher in proportion to the quantity of amphotericin B received during the week: 357 ng/mL (100 mg 4 to 5 times/week) > 263 ng/mL (50 mg 4 to 5 times/week) > 227 ng/mL (50 mg 1 to 3 times/week) (differences between Cmin were statistically non significant) (Figure 2). 

Table 1 shows the serum Cmin of 11 patients on daily regimen of amphotericin B before and after the change from D-Amph to L-AmB or following a dose increase for L-AmB. In most cases, these modifications caused an increase in Cmin, although trough levels were maintained or slightly reduced in 4/11 patients (Cmin changes were statistically non significant). Cmin was also compared in patients treated with more than one scheme of amphotericin B in an intermittent regimen during the therapeutic consolidation phase. Table 2 shows the variable response of the same patients with the progressive reduction of the quantity of amphotericin B received weekly. In some cases, a reduction in the trough levels of the drug was found; however, in 6/11 cases, the Cmin value was maintained or increased with prolonged use of amphotericin B relative to the corresponding values in the initial amphotericin B daily course. 

**TABLE 1:** Changes in the minimum concentration of Amphotericin B in serum (ng/ml serum) according to the drug formulation and daily dose administered to the patient at different times during antifungal treatment.

	A)D-Amph →	B)L-AmB	Level		A)L-AmB →	B)L- AmB	Level
Patient	30 to 50 mg/d	100 mg/d	change	Patient	50 mg/d	100 mg/d	change
**1**	137	568	+315%	**6**	71	101	+42%
**2**	284	819	+188%	**7**	79	884	+1019%
**3**	343	316	-8%	**8**	117	105	-10%
**4**	348	280	-20%	**9**	317	315	-1%
**5**	358	557*	+56%	**10**	438	765	+75%
				**11**	1.281	1.990	+55%

**D-Amph:** deoxycholate amphotericin B; **L-AmB:** liposomal amphotericin B; **A:** first therapeutic course; **B:** second therapeutic course; (*) L-AmB: 50 mg/d.

**TABLE 2:** Minimum concentration of liposomal Amphotericin B (ng/mL) in the serum of the patient receiving intermittent infusions with progressive reduction of the weekly antifungal dose after the treatment induction phase.

Patient	Therapeutic amphotericin schedule			
	Initial	L-AmB 100 mg	L-AmB 50 mg	L-AmB 50 mg
	daily course	4 to 5 times/w	4 to 5 times/w	1 to 3 times/w
1	217*	400	283	330
2	491**	88	100	666
3	495**	ND	117	124
4	520***	665	834	617
5	1,990**	339	190	106
6	252***	ND	735	922
7	394**	ND	ND	495
8	ND	ND	144	97
9	285*	ND	410	ND
10	907**	585	ND	ND
11	ND	241	228	ND
Geometric mean	475	317	261	309
CI (95%CI)	(276-819)	(163-617)	(152-451)	(173-550)
n	9	6	9	8

(*) Deoxycholate amphotericin B (only in the initial course); (**) Liposomal amphotericin B, 100 mg/day; (***) Liposomal amphotericin B, 50 mg/day. **w:** week. **ND:** not done.

During the induction phase of antifungal therapy for patients with cryptococcal meningitis, there was no difference in the median Cmin of amphotericin B between 7 patients whose outcome was death (89 to 907 ng/mL, median=252 ng/mL) and the 16 survivors (94 to 839 ng/mL, median=316 ng/mL).

The MIC of amphotericin B for the *Cryptococcus* spp. isolates from 12 patients ranged from 0.06 to 0.5 µg/mL (MIC^50^=0.125 µg/mL and MIC^90^=0.5 µg/mL, geometric mean = 0.166 µg/mL). The mean Cmin obtained with each schedule of intermittent therapy was equal or higher than the MIC of amphotericin B for the *Cryptococcus* isolates from 10/12 patients. The distribution of MIC according to the outcome of 8 patients with cryptococcal meningitis was 0.125, 0.125, 0.125, 0.25, and 0.5 µg/mL (geometric mean = 0.189 µg/mL) in 5 survivors and 0.125, 0.125, and 0.5 µg/mL (geometric mean = 0.198 µg/mL) in 3 patients who died. There were no statistically significant differences in MICs according to patient outcome.

## DISCUSSION

The present study, which was conducted under clinical conditions with non-standardized treatment, confirmed that amphotericin B maintains trough blood levels with a presumable antifungal action that lasts several days after the previous dose is received. Herein, a relationship was detected between the Cmin of amphotericin B and the therapeutic dose used; however, this was accompanied by a wide variability among patients. To reduce the cost of antifungal therapy, D-Amph was initially administered to many patients but was later replaced with L-AmB when the toxicity of the former could not be controlled by reducing the daily dose. Further, L-AmB was commonly used at a dose less than 3 mg/kg weight/day in patients previously treated with D-Amph. Although L-AmB is less toxic, its high cost has led to the use of lower doses than those recommended and usually was administered for stabilized patients requiring D-Amph discontinuation because of its toxicity^12^ ^,^ ^13^. Another particularity of the present study, related to the absence of flucytosine in Brazil, prolonged treatment with amphotericin B has been performed for patients with cryptococcal meningitis, with the aim of reaching a minimum cumulative dose of 2,000 to 3,000 mg. This became possible to determine the Cmin of amphotericin B in different schemes of non-daily use for patients after being discharged from the hospital and before the phase of maintenance with fluconazole alone.

The Cmin values for D-Amph and L-AmB were comparable to those detected in the initial studies on the pharmacokinetics of the daily administration of L-AmB^2^ ^,^ ^14^. The increase in Cmin with the increase in the daily dose of L-AmB was observed previously, despite the lack of a directly proportional relationship^15^. The blood trough levels of L-Amb were higher than those obtained with the tolerated daily doses of D-Amph; however, a significantly higher Cmin relative to that with D-Amph was only achieved with a daily dose of 100 mg (~2 mg/kg weight). In the non-daily regimen of L-AmB, the patient had measurable amphotericin B in serum even when 50 mg was infused for the seven prior days, although, mean trough serum concentration was found to decrease with a reduction in the dose and number of weekly infusions. This observation agrees with the results of the Cmin obtained in a few days after the discontinuation of L-AmB treatment, which revealed more reduced levels in patients who had received lower doses of the drug^15^ ^,^ ^16^. The blood concentrations of amphotericin B were markedly reduced 24 to 48 h after infusion, and despite the administration of a high dose of 10 mg/kg weight/week, a Cmin of less than 1 µg/mL was observed after 168 h^9^.

In addition to the wide dispersal of Cmin among patients receiving the same dose of amphotericin B, a variation was observed in the individual response to the increase in the daily infused dose. Although some patients had a non-proportional increase in Cmin, others did not increased the trough serum concentration. The variability in this response was more evident among patients receiving an intermittent regimen of amphotericin B in the consolidation treatment phase. The progressive reduction of the dose of this antifungal agent and the number of weekly infusions was followed by a decline in Cmin in some patients and unexpectedly by the maintenance or elevation of Cmin in others. The pharmacokinetics of L-AmB is more variable than that of D-Amph, as observed in different studies^14^ ^,^ ^17^. Among other possible causes, the variability in L-AmB pharmacokinetics has been attributed to the saturation of the mechanisms of clearance for liposomes containing amphotericin B, which may occur after consecutive infusions, especially with high doses^15^ ^,^ ^18^. Differences in inflammatory status and the intensity of parasitism and activation of the mononuclear phagocytic system are also probable explanations of the variability in amphotericin B levels among patients. In a murine model of *Leishmania* infection, the hepatic uptake of L-AmB was lower in infected animals than in uninfected animals^19^.

When a daily L-AmB regimen was administered, the ratio of Cmax/MIC for the isolated fungi was higher in children with a full clinical response than in those with a partial response to antifungal treatment^20^. In the present study, the Cmin of patients treated daily with D-Amph and/or L-AmB was not associated with the outcome of patients with cryptococcal meningitis; this may be due to the high susceptibility to amphotericin B of the *Cryptococcus* isolated from cryptococcosis patients at the study center^21^. When a non-daily L-AmB regimem is employed, the Cmin may be more important for the outcome if there is a longer period between infusions. It is showed that administering a weekly dose of L-AmB (i.e., 7.5 mg/kg, 10 mg/kg, or 15 mg/kg) the Cmin was deemed sufficient to act against susceptible isolates of *Candida* spp. and *Aspergillus* spp. after one week of infusion^9^ ^,^ ^16^. However, prophylaxis or the preemptive use of high L-AmB doses once per week was associated with frequent adverse effects and was not completely safe for fungemia prevention^4^ ^,^ ^22^. Comparatively, regimens for ambulatory treatment with L-AmB 50 mg or 100 mg four to five times per week yielded median Cmin values of amphotericin B that were higher than the MIC of amphotericin B for the *Cryptococcus* spp. isolates from 10/12 patients.

The present study had some limitations. Only one pharmacokinetic parameter of amphotericin B was assessed and samples were collected during non-fixed stages of antifungal therapy, which resulted in a relatively small number of samples for each therapeutic scheme. Free amphotericin B in blood, which is the fraction that diffuses to the remaining tissues, was not measured. Finally, the patients enrolled in this study were also receiving other medications, especially fluconazole and antiretroviral drugs, which were not found to have any relevant effect on the pharmacokinetics of amphotericin B.

By determining the Cmin values we could demonstrate the validity of administering the intermittent regimen with amphotericin B for consolidation treatment of systemic fungal infections, particularly cryptococcosis. Furthermore, we could suggest the frequency of use and the safest dose for administration. As some patients had a low Cmin when administered a relatively high daily doses of amphotericin B, simultaneous prescription of another antifungal agent is important during the initial treatment phase of patients with cryptococcal meningitis.
